# Brain White Matter Hyperintensity Lesion Characterization in T_2_ Fluid-Attenuated Inversion Recovery Magnetic Resonance Images: Shape, Texture, and Potential Growth

**DOI:** 10.3389/fnins.2019.00353

**Published:** 2019-04-16

**Authors:** Chih-Ying Gwo, David C. Zhu, Rong Zhang

**Affiliations:** ^1^Department of Information Management, Chien Hsin University of Science and Technology, Zhongli District, Taiwan; ^2^Department of Radiology and Psychology, and Cognitive Imaging Research Center, Michigan State University, East Lansing, MI, United States; ^3^Department of Neurology and Neurotherapeutics, Department of Internal Medicine, University of Texas Southwestern Medical Center and Institute for Exercise and Environmental Medicine, Texas Health Presbyterian Hospital Dallas, Dallas, TX, United States

**Keywords:** brain T_2_ FLAIR hyperintensity, shape, texture, potential growth, morphology

## Abstract

Prior methods in characterizing age-related white matter hyperintensity (WMH) lesions on T_2_ fluid-attenuated inversion recovery (FLAIR) magnetic resonance images (MRI) have mainly been limited to understanding the sizes of, and occasionally the locations of WMH lesions. Systematic morphological characterization has been missing. In this work, we proposed innovative methods to fill this knowledge gap. We developed an innovative and proof-of-concept method to characterize and quantify the shape (based on Zernike transformation) and texture (based on fuzzy logic) of WMH lesions. We have also developed a multi-dimension feature vector approach to cluster WMH lesions into distinctive groups based on their shape and then texture features. We then developed an approach to calculate the potential growth index (PGI) of WMH lesions based on the image intensity distributions at the edge of the WMH lesions using a region-growing algorithm. High-quality T_2_ FLAIR images containing clearly identifiable WMH lesions with various sizes from six cognitively normal older adults were used in our method development Analyses of Variance (ANOVAs) showed significant differences in PGI among WMH group clusters in terms of either the shape (*P* = 1.06 × 10^−2^) or the texture (*P* < 1 × 10^−20^) features. In conclusion, we propose a systematic framework on which the shape and texture features of WMH lesions can be quantified and may be used to predict lesion growth in older adults.

## Introduction

The presence of white matter hyperintensities (WMH) on T_2_ fluid-attenuated inversion recovery (FLAIR) magnetic resonance images (MRI) is common in older adults over 65 years old with a prevalence rate of ~ 60–80% in the general population (De Leeuw et al., 2001; Wen and Sachdev, 2004). WMH lesions are even more extensive in those with vascular or Alzheimer's disease (AD) type of dementia when compared with cognitively normal older adults, suggesting its role in dementia pathogenesis and neurocognitive dysfunction (Bombois et al., 2007; Kloppenborg et al., 2014; Lee et al., 2016). WMH is also frequently observed in patients with multiple sclerosis (MS) (Loizou et al., 2015; Newton et al., 2017). Qualitative and quantitative WMH characterization has been used as a biomarker to assist cerebrovascular and neurodegenerative disease diagnosis and to assess treatment effects (Wardlaw et al., 2013). The pathogenic mechanisms of WMH are not well-understood, and have been attributed to cerebral small vessel disease (CSVD), white matter demyelization, or both, indicating brain white matter lesions (Greenberg, 2006; Wardlaw et al., 2013). Furthermore, periventricular and subcortical deep WMHs may have different pathogenic mechanisms (Schmidt et al., 2011; Poels et al., 2012; Tseng et al., 2013).

The most commonly used methods for WMH quantification in brain aging, vascular, and AD type of dementia are to measure its regional or total volume (i.e., the sum of WMH voxel size) within the whole brain based on image tissue segmentation algorithms (DeCarli et al., 2005; Wardlaw et al., 2013). This method, however, neglects entirely the typological or morphological features of WMH lesions which may have important clinical significance as demonstrated in recent studies in patients with MS (Loizou et al., 2015; Newton et al., 2017).

WMH shape is a basic morphological feature which can be derived from T_2_ FLAIR images after tissue segmentation. Shape feature extraction, recognition, and classification can be implemented either in the original or the transformed image space (Khotanzad and Hong, 1990; Mikolajczyk et al., 2003; Carmichael and Hebert, 2004; Tahmasbi et al., 2011). Current shape classification methods include mainly the following: (1) one-dimensional function shape representation (Kauppinen et al., 1995; Yadav et al., 2007; Zhang and Lu), (2) polygonal approximation (ShuiHua and ShuangYuan), (3) spatial interrelation feature (Sebastian et al., 2004; Guru and Nagendraswamy, 2007; Bauckhage and Tsotsos), (4) moments (Mukundan, 2004; Celebi and Aslandogan; Taubin and Cooper), (5) scale-space methods (Zhang and Lu, 2003; Kpalma and Ronsin, 2006), and (6) shape transform domains (Chen and Bui, 1999; Zhang and Lu). These methods for shape classification may be suitable for specific applications in various fields but have major limitations for shape characterization of brain lesions. For example, method (1) is highly sensitive to noise, and inaccurate boundary definition can cause large errors; method (2) can only represent the object appearance but not all shape features; method (3) may be used to describe the general appearance of an object, but is limited by the orientation and size of the object; method (4) contains redundant information in the image feature vectors and thus unique images cannot be reconstructed back; method (5) is limited to the shapes which have shallow concavities/convexities; and method (6) requires the definition of the shape contour starting point derived from other methods. In order to characterize the complex shapes of brain lesions, a technique needs to be invariant to the orientation of a lesion, be resistant to image noise and be able to define a one-to-one relationship between feature vector and shape. In this regard, Zernike transformation can satisfy these criteria (Khotanzad and Hong, 1990). Similar to Fourier analysis, shape features of an object captured on MRI can be represented by the coefficients of shape function of the Zernike polynomial expansion (i.e., Zernike transform), referred to as Zernike moments (ZMs) (Zernike, 1934). In this study, we applied Zernike transformation to extract WMH shape features for pattern recognition and classification in cognitively normal older adults.

Image texture is another morphological feature which can be categorized through modeling (Chen et al., 1989), structure (Chow and Rahman, 2007), transformation (Tsai and Hsiao, 2001), and statistics based methods (Haralick et al., 1973; Iivarinen et al., 1996). Model-based and structure-based methods work best for repeating texture patterns but are not suitable for irregular texture patterns such as those in brain lesion images. The transformation based method works best in identifying sub-regions with known characteristics, but does not work well on unknown and potentially complicated patterns such as those in brain lesion images. Statistical-based methods describe the texture in the distribution of and relationships between gray-level values in an image. These statistics-based methods can normally describe objects better than the structure and transformation based methods because they are invariant to the orientation, the size of an object, and also robust to the noise inside the object (Castellano et al., 2004). Since WMH lesions often have various sizes, orientations, and locations, and are manifested across multiple image slices, a statistics-based method is likely to be the best choice to accommodate these complexities. Therefore, we adopted a statistical method based on fuzzy logic to construct the image intensity histogram of WMH lesions for texture feature extraction.

Finally, we have thought that as a potential imaging biomarker of brain aging and CSVD, the size, shape, and image texture of WMH lesion may change with time (Sachdev et al., 2007; Godin et al., 2011) which may reflect the progression of the underlying pathological process. In this regard, recent studies have shown that the immediate surrounding areas of clearly defined WMH lesions may be at risk for further tissue damage and conversion to lesions (Maillard et al., 2014; Promjunyakul et al., 2016). These areas are classified as WMH penumbras (Maillard et al., 2014). To characterize WMH lesions as well as their penumbras, we developed a seed-based region-growing algorithm to characterize WMH boundaries to explore the potential growth of WMH lesions. We defined this specific WMH boundary characteristic as potential growth index (PGI). To explore whether the shape and texture characterization techniques can potentially be used to predict lesion growth, we assessed whether different shape and texture patterns are related to PGI.

## Methods and Results

### MRI Acquisition

Full-brain 2D T_2_ FLAIR images were collected on a Philips Achieva 3T scanner (Philips Healthcare, Best, the Netherlands) with the following parameters: axial, time of echo (TE) = 125 ms, time of repetition (TR) = 11 s, time of inversion (TI) = 2,800 ms, field of view (FOV) = 23 cm × 23 cm, slice thickness = 2.5 mm, number of slices = 64 with no gaps, acquisition matrix size = 352 × 212, and reconstructed matrix size = 512 × 512. All subjects signed informed consent approved by the Institutional Review Boards of the UT Southwestern Medical Center and Texas Health Presbyterian Hospital of Dallas. Six T_2_ FLAIR brain image datasets (two male, four female, 75 ± 4 years old and normal cognition), which contained clearly identifiable white matter hyperintensity (WMH) lesions with various sizes, were selected from an healthy aging study we published previously (Tarumi et al., 2014).

### T_2_ FLAIR Image Segmentation

**Figure 1:**
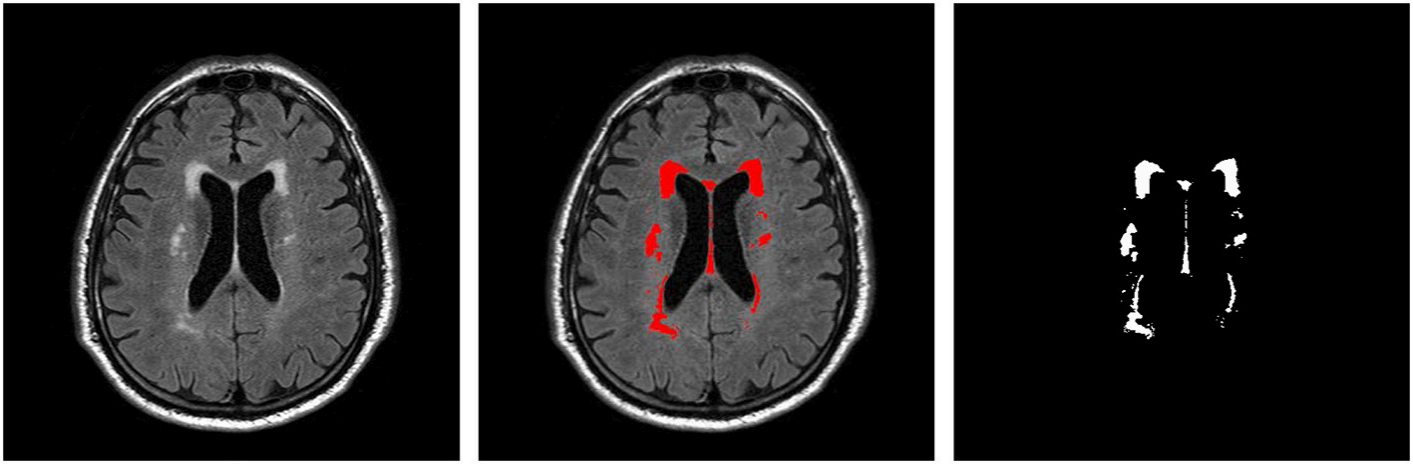
An example T_2_ FLAIR image from one subject showing multiple white-matter hyperintensity (WMH) lesions; the results of WMH segmentation using the lesion prediction algorithm (LPA) showing in red, and a WMH binary mask after tissue segmentation, which was used in shape feature extraction.

T_2_ FLAIR WMH regions were segmented on each 2D image through the lesion prediction algorithm (LPA) implemented in the Lesion Segmentation Toolbox (LST) version 2.0.12 for Statistical Parametric Mapping (SPM12). In LPA, the algorithm is trained using a logistic regression model on T_2_ FLAIR brain images from 53 MS patients with severe lesion patterns. LPA was also validated in other patient populations such as older adults with diabetes (Maldjian et al., 2013). The fitness of a new T_2_ FLAIR brain image to this model provides an estimate of lesion probability for each voxel in the image. In this study, we used a threshold of 0.5, as suggested by LST, on the obtained lesion probability maps to identify WMH regions. The segmentation accuracy was further verified through visual inspection. Figure 1 shows an example of the segmentation.

### Lesion Size Distribution

**Figure 2:**
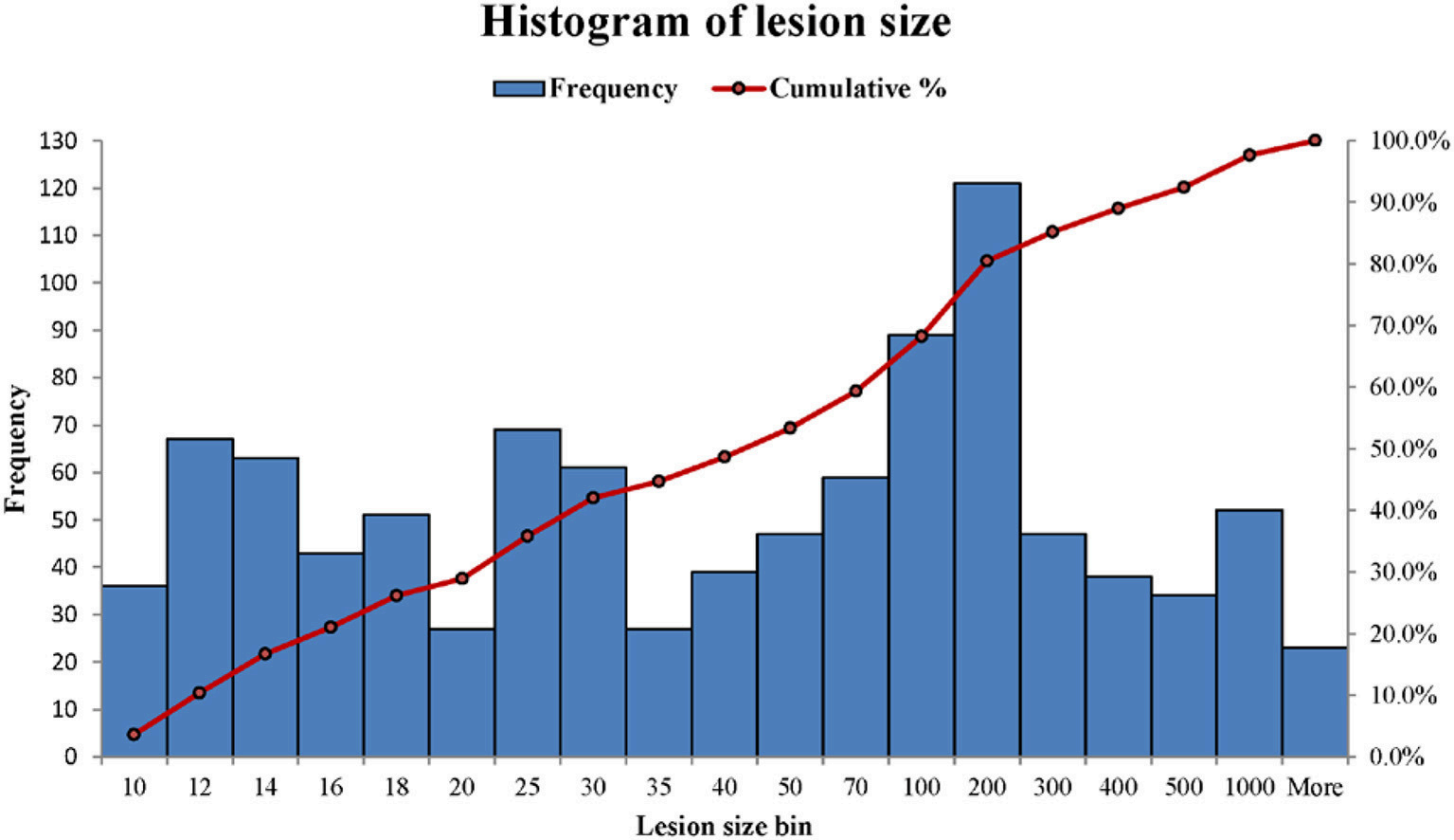
The histograms of WMH lesion size distributions in two representative subjects. The lesion size bin of 50 represents the lesion size range from 40 × 40 to 50 × 50 voxels. The frequency scale (the left vertical axis) is the counts the number of lesion sizes within a lesion size bin. The cumulative percentage of lesion size relative to the total lesion counts is labeled on the right vertical axis.

WMH binary masks generated from 2D T_2_ FLAIR images (Figure 1) were used to obtain WMH size distribution. To minimize artifacts, only those masks with more than 10 connected WMH voxels (voxel size: 0.45 mm × 0.45 mm) on an image were considered probable lesions and were used for further characterization, which resulted in a total of 993 WMH lesions. Fitting each of these lesions within a square, these lesions had a size range of from 6 × 6 to 176 × 176 voxels. The lesion size distributions of six subjects are shown in Figure 2. Of note, more than 93% of these lesions are ≤ 60 × 60, and only about 1.5% are ≥ 120 × 120 voxels.

### WMH Shape Feature Extraction and Classification in 2D

#### WMH Shape Feature Extraction Using Zernike Transformation

Zernike transformation has been used extensively in imaging shape feature extraction and pattern recognition (Papakostas et al., 2007; Wee and Paramesran, 2007). The coefficients of Zernike polynomial expansion of an object are referred to as Zernike moments (ZMs) which are used to represent the shape features of analyzed objects. In this study, Zernike polynomials were expressed in polar coordinates defined on a unit disc, which are a complete set of orthogonal basis functions (Papakostas et al., 2007; Wee and Paramesran, 2007). The lower-order ZMs describe global contour and gross shape features, and the higher-order ZMs describe regional and fine topological details of a shape (Gwo and Wei, 2016). Of note, the magnitudes of ZMs are not only rotational invariant but also robust to small perturbations on the contour of a shape image (Teh and Chin, 1988).

For a 2D image object (a WMH lesion image segmented from a T2 FLAIR image in this work), using polar coordinates, the complex Zernike moments of order n with repetition m can be represented as the inner product of a shape function *f* (*r*, θ) with the basis function of Zernike polynomials, *V*_*nm*_ (*r*, θ), specifically as

(1) $$\begin{array}{r} Z_{nm}=\frac{n+1}{\pi }\underset{0}{\overset{2\pi }{\int }}\underset{0}{\overset{1}{\int }}f\left(r,\theta \right)V_{nm}^{*}\left(r,\theta \right)rdrd\theta ,\;|r|\leq 1, \end{array}$$

where $V_{nm}^{*}\left(r,\theta \right)$ denotes the complex conjugation of *V*_*nm*_(*r*, θ). The basis function of Zernike polynomial is given by

(2) $$\begin{array}{r} V_{nm}\left(r,\theta \right)=R_{nm}\left(r\right)e^{im\theta },\;i=\sqrt{-1} \end{array}$$

where the radial polynomial, *R*_*nm*_(*r*), is defined as follows:

(3) $$\begin{array}{r} R_{nm}\left(r\right)=\overset{\frac{n-|m|}{2}}{\underset{k}{\sum }}{\left(-1\right)}^{k}\frac{\left(n-k\right)!}{k!\left(\frac{n+|m|}{2}-k\right)!\left(\frac{n-|m|}{2}-k\right)!}r^{n-2k} \end{array}$$

where 0 ≤ |*m*| ≤ *n*, *n*− |*m*| is an even integer, and *n*≥0.

**Figure 3:**
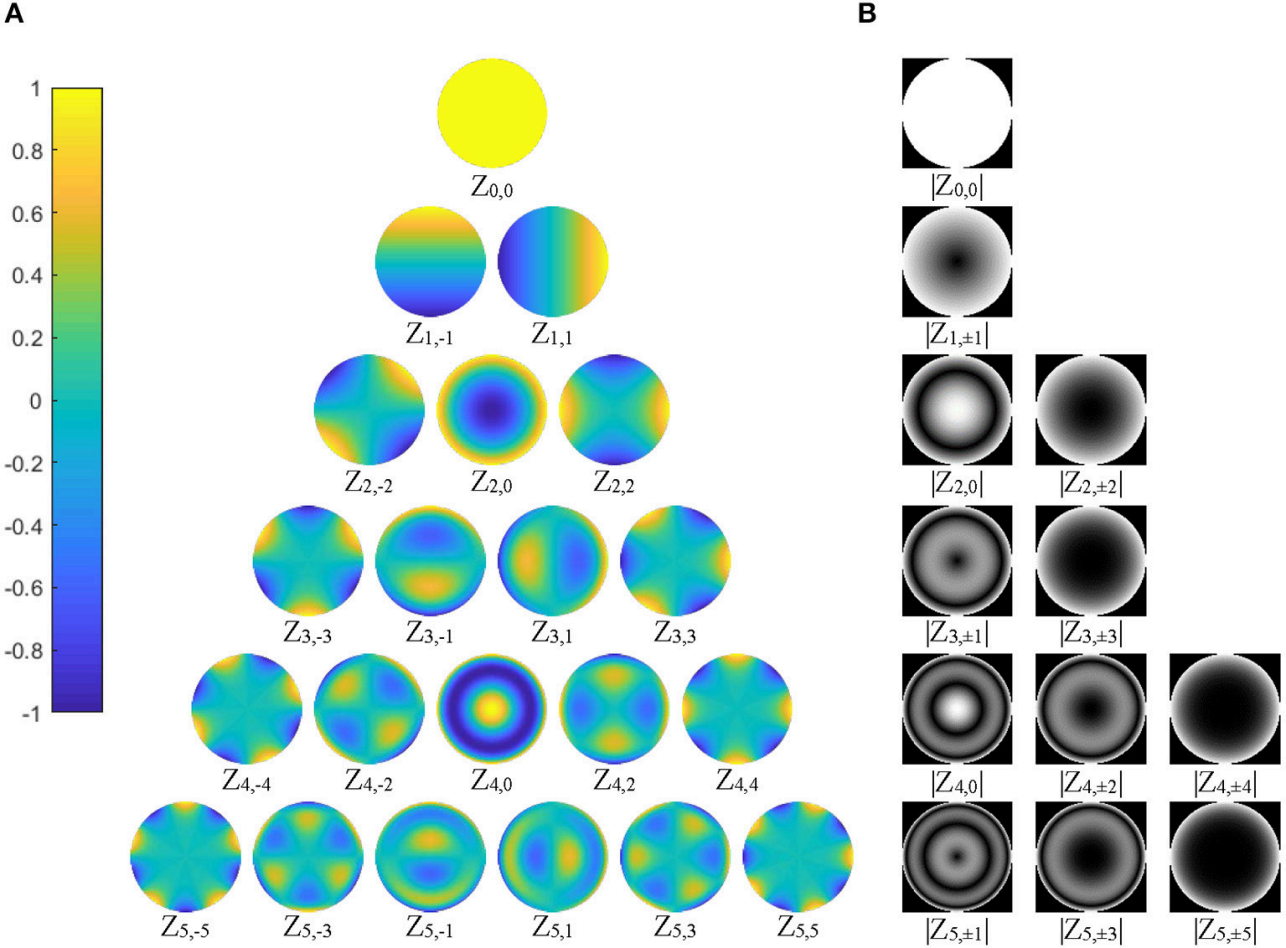
**(A)** The 21 basis functions of Zernike polynomials, *V*_*nm*_(*r*, θ), with order *n* ≤ 5, are illustrated. The polynomials have a radial range of [−1, 1] (|*V*_*nm*_(*r*, θ)| ≤ 1), shown by the color bar on the left column; **(B)** 12 distinctive magnitude images, which are rotational invariance, are shown, corresponding to the polynomials in **(A)**.

Since the shape features represented by ZMs at orders higher than six are usually too small (small ZM magnitude) to be detected reliably by human eyes (Charman, 2005), the maximum Zernike transformation order was set to five in this study (Figure 3). In Zernike transformation, |*V*_*n*, +*m*_(*r*, θ)| = |*V*_*n*, −*m*_(*r*, θ)|, and |*Z*_*n*, +*m*_| = |*Z*_*n*, −*m*_|. The number of distinctive ZM magnitudes for an expansion up to order *n* is computed as follows:

(4) $$\{\begin{array}{l} \begin{array}{l} {\left(\frac{n+2}{2}\right)}^{2}\text{ if ordern is even}\\ \frac{\left(n+3\right)\left(n+1\right)}{4}\text{ if order n is odd} \end{array} \end{array}$$

**Figure 4:**
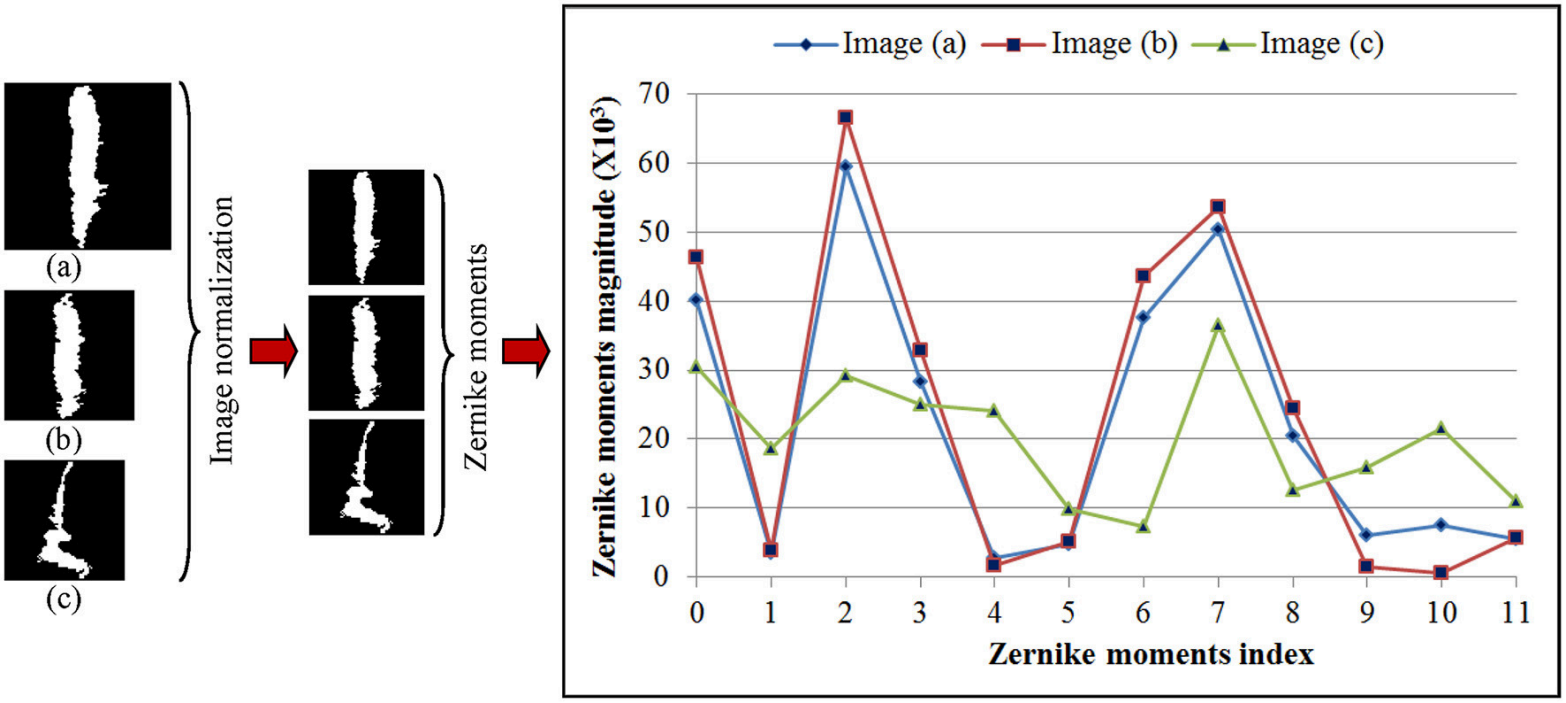
Three representative white mater hyperintensity masks generated after tissue segmentation with different sizes and shape are shown in the left column. The images are normalized to the same size of 60 × 60 voxels shown in the middle column. The magnitudes of 12 Zernike moments coefficients based on ZM orders ≤5 of Zernike polynomial expansion are shown in the right column for comparison.

Shape feature extraction procedures based on Zernike transformation are illustrated in Figure 4. In this illustration, we chose three WMH masks, two with a similar shape but different sizes, and one with both different shape and size. To simplify computation complexity, these image masks with different sizes were first scaled to the same size of 60 × 60 voxels so that the ZM magnitudes can be compared on a same scale. Each *Z*_*nm*_ was calculated using Equation (1–3). The calculation resulted with 21 ZM complex coefficients with maximum order *n* = 5. Based on the magnitudes of the ZM coefficient, only 12 coefficients were needed to extract shape features since the WMH shape (mask) generated with tissue segmentation is rotational invariant. As shown in the right column of Figure 4, the two lesion images (a) and (b) with a similar shape have similar ZMs magnitudes at all 12 coefficients. On the contrary, the ZM magnitudes of WMH lesions with a different shape are different from the other two.

#### WMH Shape Classification

**Figure 5:**
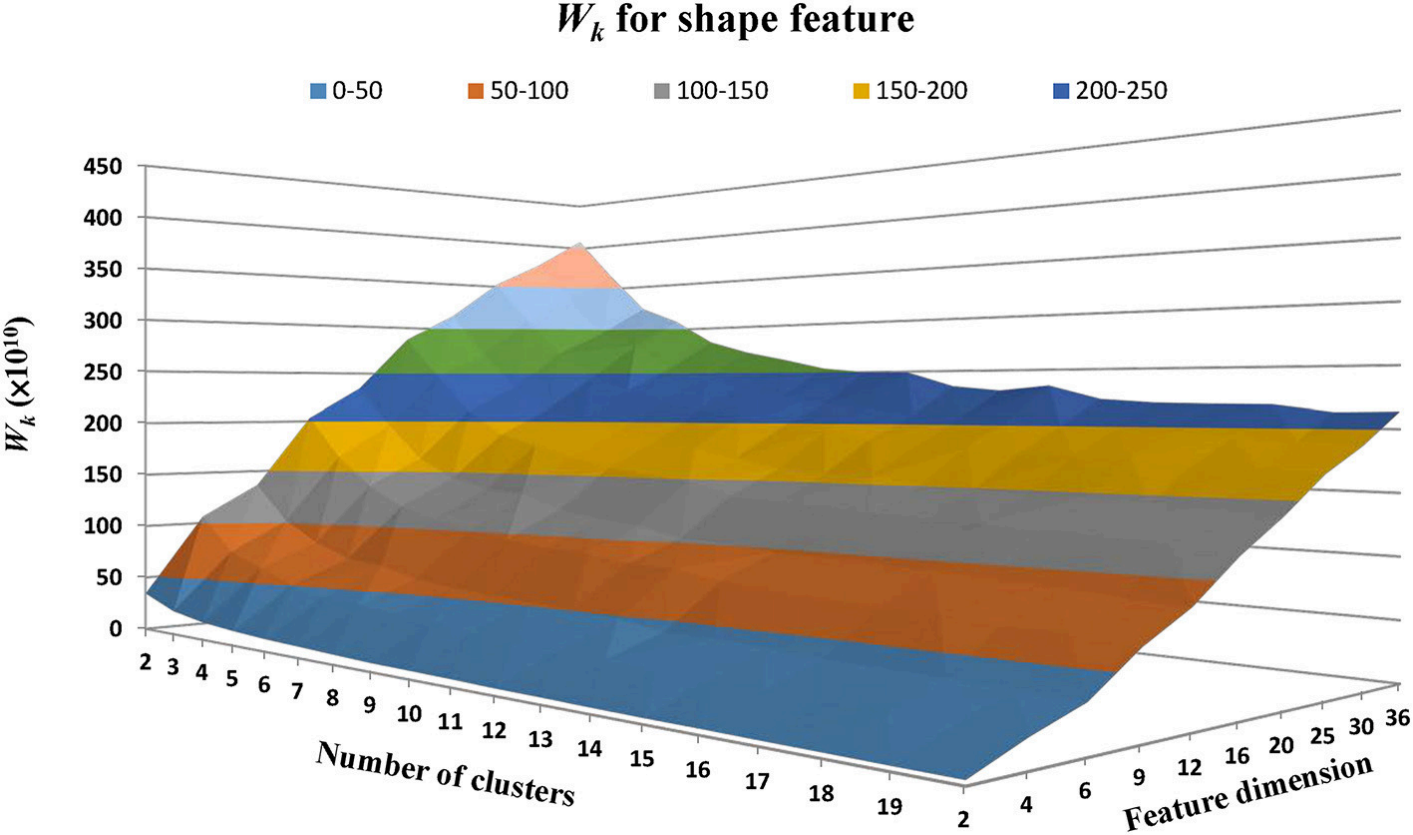
The within-cluster dispersion *W*_*k*_ as a function of the number of shape clusters and feature dimensions. The feature dimensions are the number of distinctive magnitudes of the ZMs. *W*_*k*_, in general, tends to decrease with the number of clusters but increase with the number of feature dimensions.

We then classified the lesion images to different clusters (groups) based on the similarity on shape features. Current common clustering algorithms, such as the K-means clustering algorithm, requires data-specific a priori selection on the number of clusters (Zhao, 2012). For instance, if the number of clusters is too small, the WMH lesion images with noticeable different shapes may be grouped inappropriately into a same cluster. On the other hand, if the number of clusters is too large, lesion images with trivial differences may be assigned into different clusters, confounding potential clinical significance. Finding the appropriate number of clusters using model simulation is one way to resolve this dilemma (Zhao, 2012). However, this procedure has to be carried out for all choices of shape feature dimensions. To simplify the procedures, the estimation of cluster characteristic indices based on sum of within-cluster dispersions [*W*_*k*_ in Equation (5)] or its variants were proposed (Ball and Hall, 1965; Calinski and Harabasz, 1974; Xu, 1997; Tibshirani et al., 2001). For a better understanding of the influences of the classifiable number of clusters and the feature dimensions derived from the Zernike transform on *W*_*k*_, we plotted *W*_*k*_ as a function of cluster numbers and feature dimensions which are equivalent to the numbers of the distinctive magnitudes of the ZMs (Figure 5). The ZMs for WMH shapes from one to 10 orders were calculated to generate 2 to 36 dimensional feature vectors (Equation 4). Euclidean distance was then calculated to assess the similarity between the feature vectors. The K-means clustering algorithm was applied for grouping purpose. *W*_*k*_ was calculated based on the 2 to 20 cluster groups at each feature dimensions. *W*_*k*_, in general, decreases with the increase of the number of clusters but increases with the increase of the number of feature dimensions (Figure 5). For a specified feature dimension, a better grouping result is likely achieved at a lower *W*_*k*_ value by finding a local minimum. However, in some feature dimensions, a local minimum cannot be found even after *W*_*k*_ decreases to nearly constant. For example, when two is selected as the feature dimension, the *W*_*k*_ value remains small even at small number of clusters because two-dimensional feature vectors only represents the gross global contour and thus cannot differentiate shapes with enough details. Therefore, selecting an appropriate feature dimension is also crucial and will be discussed later. Nevertheless, once a feature dimension is selected [which was selected to be 12 in this study based on our exploration of data features (Figures 4, 5)], the optimal number of clusters can be determined based on the estimation of cluster characteristics discussed below (Desgraupes, 2013).

The distance between two points in a share feature vector space can be calculated based on Euclidian distance. The overall distance of all points in a cluster to their mean indicates the compactness of a cluster, or within-cluster dispersion. To determine the optimal number of shape clusters for WMH shape classification, we then employed a gap statistics method proposed by Tibshirani et al. (2001). This method estimates the optimal number of clusters by comparing the logarithm of the sum of within-cluster dispersions of a set of clusters to that from the reference datasets created through sampling uniformly at random from the original dataset. The sum of all within-cluster dispersions decreases gradually with the increase of number of clusters but becomes nearly constant at some points as demonstrated in Figure 5. This is so called “elbow” phenomenon, which has been used to find the optimal number of clusters (Tibshirani et al., 2001). The algorithm used to estimate the optimal number of WMH shape clusters based on the gap statistic is presented below:

1. Group the shape vectors by varying the number of shape clusters from *k* = 1, 2, …, *N* (pre-defined as the maximum to evaluate), and compute the sum of the within-cluster dispersion *W*_*k*_ for each choice *k*.
  (5) $$\begin{array}{r} W_{k}=\overset{k}{\underset{r=1}{\sum }}\underset{x_{i}\in C_{r}}{\sum }{\left(x_{i}-{\bar{x}}_{r}\right)}^{2} \end{array}$$
  where *x*_*i*_ is a data point, *C*_*r*_ denotes cluster *r*, and ${\bar{x}}_{r}$ is the vector mean of *C*_*r*_.
  Generate reference datasets (total number = *B*) by sampling uniformly at random from the original dataset within its distribution ranges of all dimensions. Although a better statistically randomness is likely achieved with a large *B*, the choice of *B* is bounded by computation demand. For each reference dataset *b*, we can generate k clusters, and we can calculate the sum of the within-cluster dispersion *W*_*kb*_ for each *k* based on Equation (5) above, where *b* = 1, 2, …, *B*; *k* = 1, 2, …, *N*. The gap statistics for each k is calculated as below:
  (6) $$\begin{array}{r} Gap\left(k\right)=\frac{1}{B}\overset{B}{\underset{b=1}{\sum }}\text{log}\left(W_{kb}\right)-\text{log}\left(W_{k}\right) \end{array}$$
2. let$l=\left(1/B\right)\underset{b}{\sum }\text{log}\left(W_{kb}\right)$, compute the standard deviation
  (7) $$\begin{array}{r} sd_{k}={\left[\frac{1}{B}\overset{B}{\underset{b=1}{\sum }}{\left(\text{log}\left(W_{kb}\right)-l\right)}^{2}\right]}^{1/2} \end{array}$$
  Let $s_{k}=sd_{\text{k}}\sqrt{\left(1+1/\text{B}\right)}$. Choose the optimal number of shape clusters *k*_opt_ by Equation (8)
  (8) $$\begin{array}{r} k_{opt}=\text{smallest }k\;\text{such that Gap}\left(k\right)\geq Gap\left(k+1\right)-s_{k+1} \end{array}$$
  In the gap statistic procedure above, *N* is a pre-selected number of shape clusters such that *k*_opt_ can be determined in the range of [1, *N*]. *B* is selected such that the value of *sd*_*k*_ converges. In this study, *N* and *B* were set to 20 and 10, respectively.

**Figure 6:**
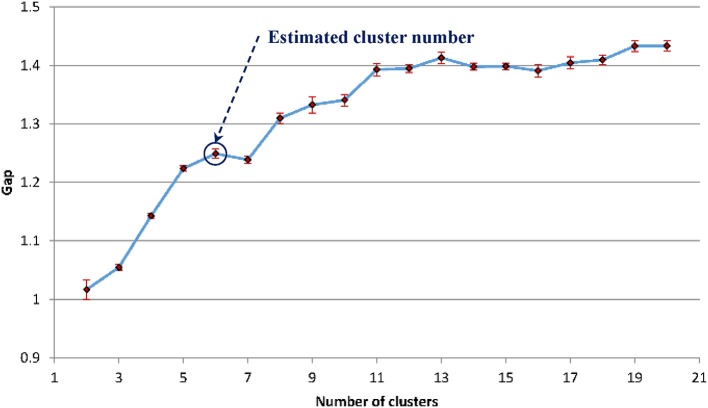
The estimated gap statistic Gap as a function of shape cluster number k (dots and solid curve), error bars are ±*s*_*k*_.

For the WMH shape datasets in this study, based on the previous discussion and Figure 5, the “elbow” phenomenon was sufficiently noticeable when the feature dimension was set to 12. At this feature dimension, only ZM magnitudes corresponding to ZM orders of *n* = 0 to 5 were used in clustering [cf. Equation (4)]. The Gap values were calculated and displayed in Figure 6; the optimal number of shape clusters was selected to be six according to Equation (8).

**Figure 7:**
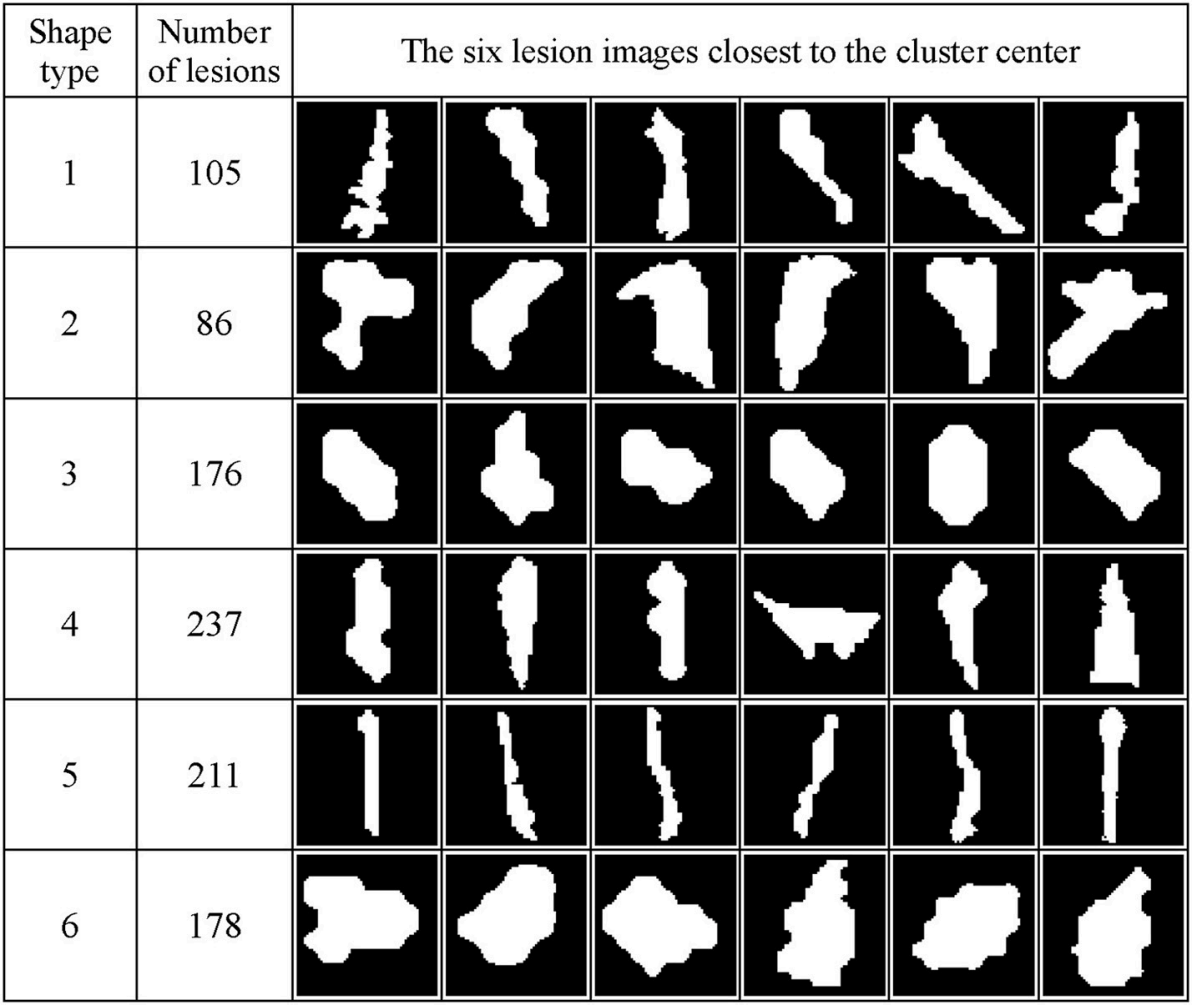
WMH shape classification results using the K-means algorithm based on the cluster number of six and feature dimension of 12. The number of lesion images in each cluster and the six normalized lesion images closest to the cluster mean in each cluster are shown. All lesion images shown in the figure were normalized to the size of 60 × 60 voxels.

Figure 7 shows the WMH shape classification results using the K-means algorithm based on the cluster number of six and feature dimension of 12. Unique shape difference can be visualized between the six clusters.

### WMH Texture Feature and Classification

#### Texture Feature Extraction

Image texture characterizes the voxel signal intensity distribution patterns in a WMH region. Statistics-based methods quantify the distribution and relationships of voxel signal values in an image region. These methods often provide better discrimination indexes than structure and spectral transformation based methods (Castellano et al., 2004).

**Figure 8:**
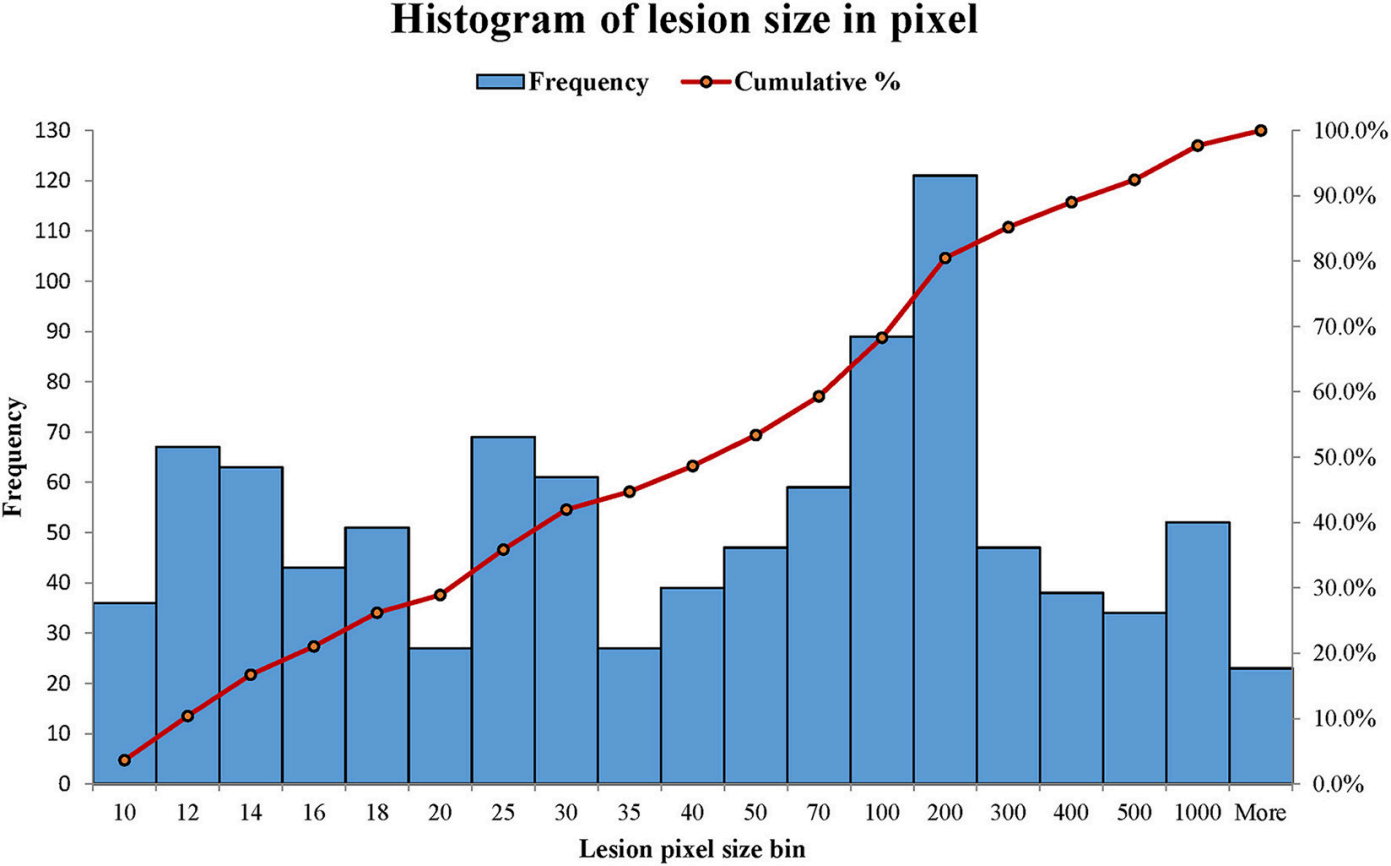
The distributions of WMH lesion size measured in number of voxels from the six subjects. A number shown in a lesion size bin (the horizontal axis) represents a lesion size range. For example, lesion size bin of 50 represents the lesion size range of 40 × 40 to 50 × 50 voxels. Frequency (the vertical axis) counts the number of lesion sizes at the lesion size bins.

WMH lesions often have various sizes, orientations and locations, and manifest across multiple image slices. In this study, the distributions of WMH lesion size measured in the number of voxels are presented in Figure 8.

Most lesions are small, with 48.64% of lesions ≤ 40 voxels (8 mm^2^ on a slice). Therefore, a robust texture analysis method needs to satisfy three requirements: (1) Texture feature should be independent of lesion orientation and location; (2) texture feature should be able to quantify small lesions, and (3) texture characterization needs to go beyond a single image slice. A statistics-based method for WMH texture feature extraction is described next.

Since WMH lesions manifests across multiple slices, we used the “WMH3D” term to emphasize the 3D perspective. Specifically, if a WMH lesion image in a slice connects directly either above, below, or diagonally to another WMH lesion image in an adjacent slice, we treat these lesion images belonging to the same lesion, called it a “WMH3D” for texture analysis. This treatment also reduces the chance of false positive in lesion identification. To characterize the voxel signal intensity distribution, potential voxel spike noise, which is often seen in images, needs to remove first. This can be accomplished by setting the voxel intensities within the boundaries of above or below three standard deviations of the mean values. To reduce the slice variation in signal intensity, a min-max normalization was applied to a WMH3D to normalize its voxel intensity based on the equation,

(9) $$\begin{array}{r} s\left(x,y,z\right)=\frac{f\left(x,y,z\right)-gMin}{gMax-gMin} \end{array}$$

where *f* (*x, y, z*) is the intensity of voxel (*x, y*) at the *z*th slice and *s*∈ [0,1], *gMax* = *Max*(voxel intensities of WMH3D) and *gMin* = *Min* (voxel intensities of WMH3D).

**Figure 9:**
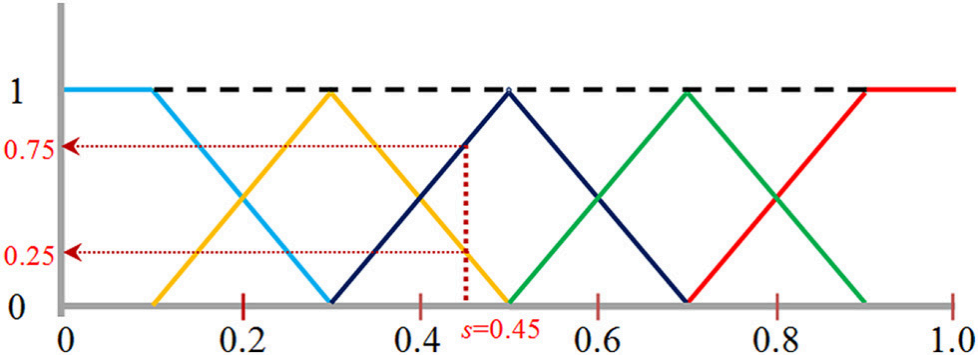
The fuzzy logic functions used for assigning voxels to five bins: bin [0, 0.2] shown in blue, bin [0.2, 0.4] in orange, bin [0.4, 0.6] in black, bin [0.6, 0.8] in green, and bin [0.8, 1.0] in red. A normalized image intensity value is assigned to its two neighboring bins based on these assignments functions. For example, s of 0.45 is assigned to a frequency value of 0.25 to the bin [0.2, 0.4], and 0.75 to the bin [0.4, 0.6] as indicated by the vertical and horizontal dotted lines.

For feature extraction, the normalized data were quantized into one of the pre-selected bins to create a histogram that represents voxel intensity distribution of a WMH3D. To minimize the interference of image noise to the frequency histogram, we propose a fuzzy logic method (Gwo and Wei, 2013) to allocate voxel intensity values to each of the pre-selected bins. Specifically, a normalized voxel intensity *s* is assigned proportionally two values, called fuzzy values, to the two neighboring bins according its relative positions to the bin centers (Figure 9). The fuzzy logic method not only is able to characterize the local image signal intensity distribution of a lesion, but also its global distribution, producing different histogram skewness based on the intensity mean value.

The fuzzy logic functions used for assigning voxels to the frequency histogram are presented in Equation (10). The fuzzy value v[*j*] at bin *j* is calculated as:

(10) $$\left\{ \begin{array}{l} v\left[0\right]=1\text{ if }s\leq \frac{1}{2n}\\ \begin{array}{l} \text{​​}v\left[j-1\right]=\frac{2j+1}{2}-s\times n\\ \text{​​}v\left[j\right]=s\times n-\frac{2j-1}{2} \end{array}\}\text{if }s\leq \frac{2j+1}{2n}\\ \begin{array}{l} \text{​​}v\left[j\right]=\frac{2j+3}{2}-s\times n\\ \text{​​}v\left[j+1\right]=s\times n-\frac{2j+1}{2} \end{array}\}\text{if }s>\frac{2j+1}{2n}\\ v\left[n-1\right]=1\text{ if }s\geq 1-\frac{1}{2n} \end{array}\right.$$

where *n* = the total number of bins, and *j* = 0, …, *n*-1. To choose a proper number of bins, there are two considerations: (1) When the number of bins increases, the accumulated fuzzy values in some bins become sparse, especially for small size lesions. Sparsity is problematic for any statistical analysis method (Hughes, 1968). The amount of data needed to obtain a reliable statistical result grows exponentially with the number of bins (Hughes, 1968); (2) conversely, if the number of bins is too small, image features may not be differentiated effectively. In this study, to facilitate WMH texture feature classification discussed below, we selected five bins for texture feature extraction.

**Figure 10:**
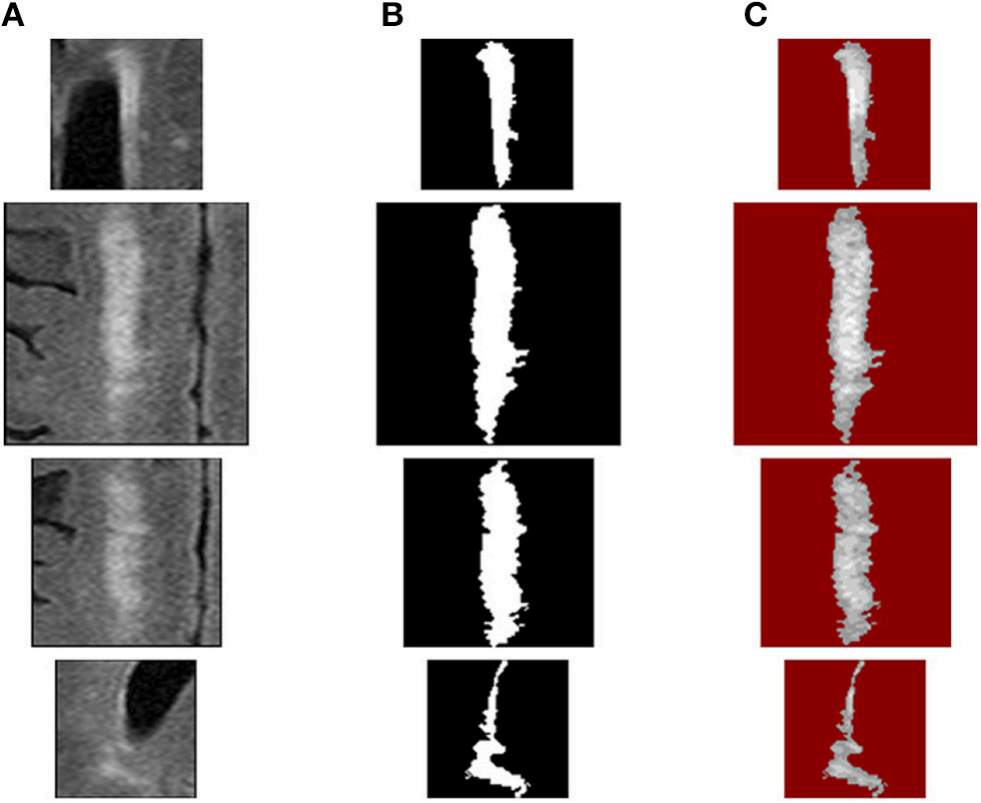
WMH texture feature extraction procedures: **(A)** source WMH lesion images, **(B)** WMH lesion mask images, and **(C)** WMH texture quantized images using fuzzy logic method.

Since the sizes of WMH lesions vary in a wide range (Figure 8), the image intensity frequency distribution histograms need to be further normalized before they can be compared. Herein, each histogram is normalized to have a total accumulative frequency of 1. For example, for a WMH lesion shown in the first row in Figure 10, the original distributions of histogram with five bins and 814 voxels would produce a texture feature vector of (266.6, 240.2, 153.9, 125.3, 28.0). To compare with other WMH lesions with different sizes, this vector was divided by 814 to become the normalized distribution of (0.3275, 0.2951, 0.1891, 0.1539, 0.0343).

#### WMH Texture Feature Classification

**Figure 11:**
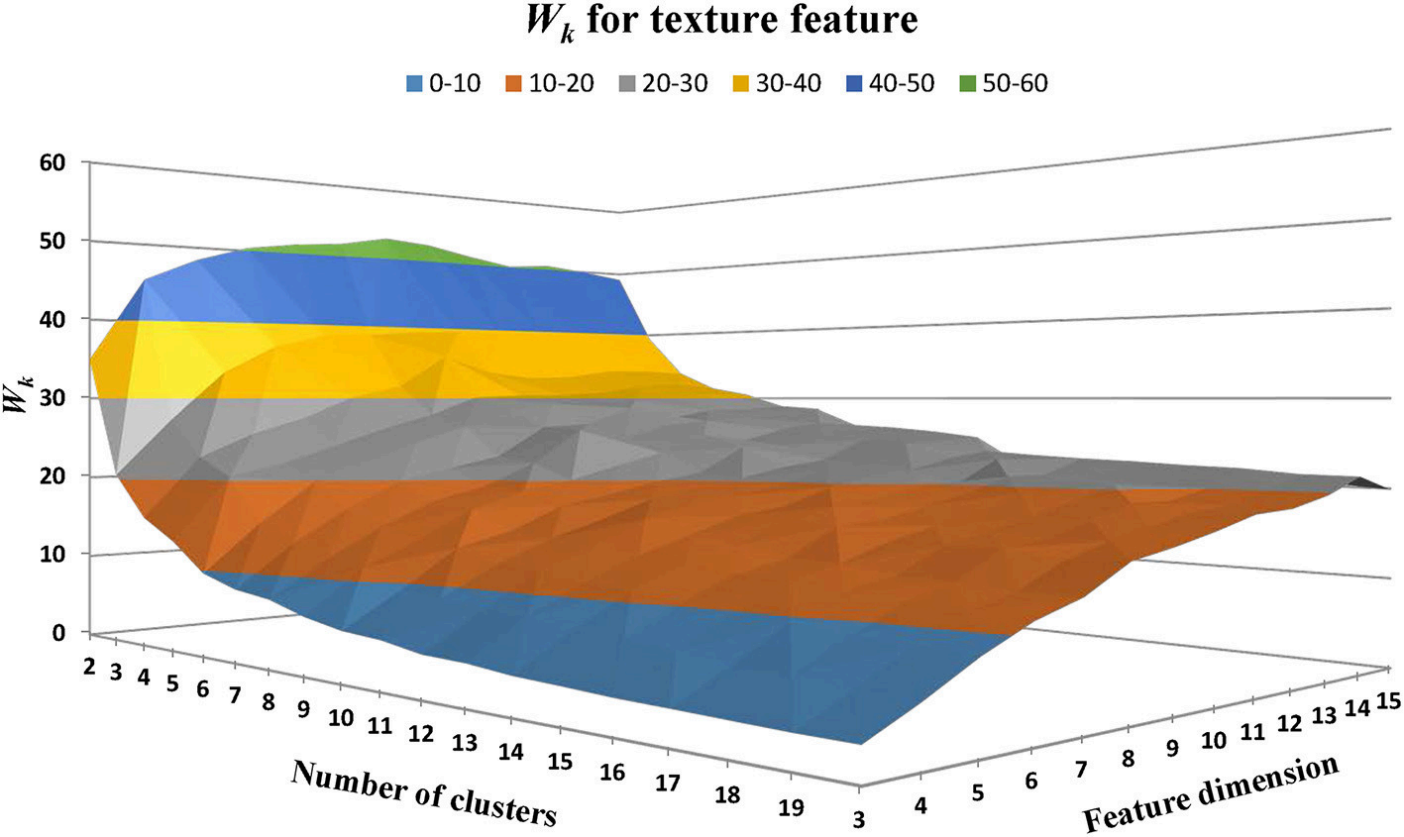
The within-cluster dispersion *W*_*k*_ as the function of the number of texture cluster and feature dimension. Note that a noticeable “elbow” phenomenon presents for a wide range of texture feature dimensions from 3 to 15.

Texture feature classification of individual WMH lesion images was conducted using a feature vector clustering method similar to those discussed above in the section of “WMH Shape Classification.” Of note, the texture feature vector is based on the histogram presented above using the fuzzy logic method. The influences of different texture feature dimensions (i.e., the number of bins used to construct the intensity histogram) and the numbers of clusters on texture feature classification were explored using the same strategy discussed above for WMH shape feature classification. Based on prior works (Shapiro and Stockman, 2001), Manhattan distance is a more preferred choice over Euclidean distance in accessing the similarity between feature vectors described in histograms. Thus, Manhattan distance was used to assess the similarity between the texture feature vectors in our work. The sum of within-cluster dispersion *W*_*k*_ value was calculated with the cluster number from 2 to 20 and the feature dimensions from 3 to 15. As illustrated in Figure 11, *W*_*k*_ tends to decrease with the increase of the cluster numbers. A noticeable “elbow” phenomenon was seen for a wide range of texture feature dimensions from 3 to 15.

**Figure 12:**
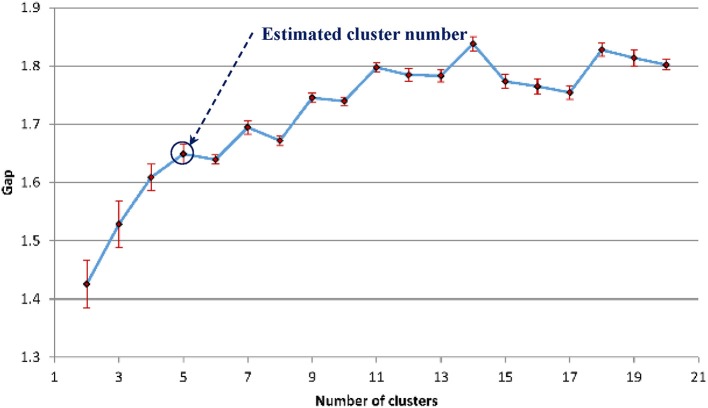
The estimated gap statistic Gap as a function of texture cluster number k (dots and solid curve), error bars are ±*s*_*k*_.

The gap statistics discussed above was applied to determine the optimal number of texture feature cluster for pattern recognition based on the K-means algorithm for grouping (Hartigan and Wong, 1979). Figure 12 shows that five is the optimal number of cluster.

**Figure 13:**
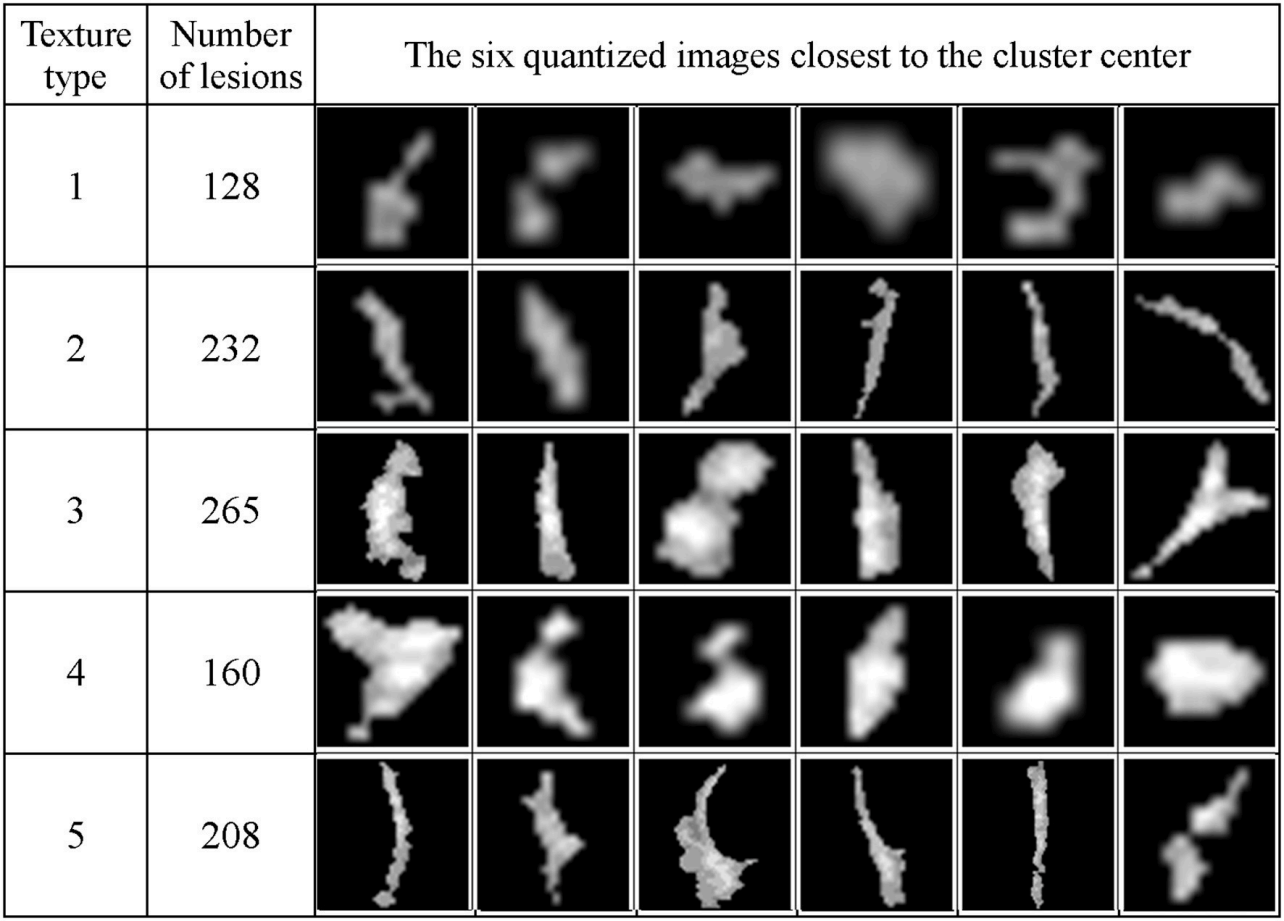
The WMH lesion images from six subjects were classified to five clusters based on their texture features. The six lesion images closest to their cluster means based on the Manhattan distance are shown for each cluster.

Figure 13 shows the texture classification results, demonstrating five unique clusters.

### WMH Potential Growth Index in 2D

We developed a seed-based region-growing algorithm to characterize WMH boundary conditions in order to explore potential growth of WMH lesions (Maillard et al., 2014; Promjunyakul et al., 2016). We hypothesized that the area of potential growth of WMH lesions has similar signal intensity as WMH lesions and is located around the boundary of WMH lesions. With a pre-defined signal intensity threshold, calculated by the extreme values in the WMH3D [Equation (9)], we can use a seed-based region-growing algorithm to find the “potential growth” voxels around the WMH boundary. The region-growing algorithm is initiated by selecting the WMH mask boundary voxels as the growing seeds. At each growing seed voxel, the eight connected neighbor voxels, defined as *A*_8_(*x, y*) in Equation (11) below, are examined iteratively until no more voxels meet a given criterion in signal intensity.

(11) $$\begin{array}{r} A_{8}\left(x,y\right)=\left\{\begin{array}{c} \left(x-1,y-1\right),\left(x,y-1\right),\left(x+1,y-1\right),\left(x-1,y\right)\\ \left(x+1,y\right),\left(x-1,y+1\right),\left(x,y+1\right),\left(x+1,y+1\right) \end{array}\right\} \end{array}$$

**Figure 14:**
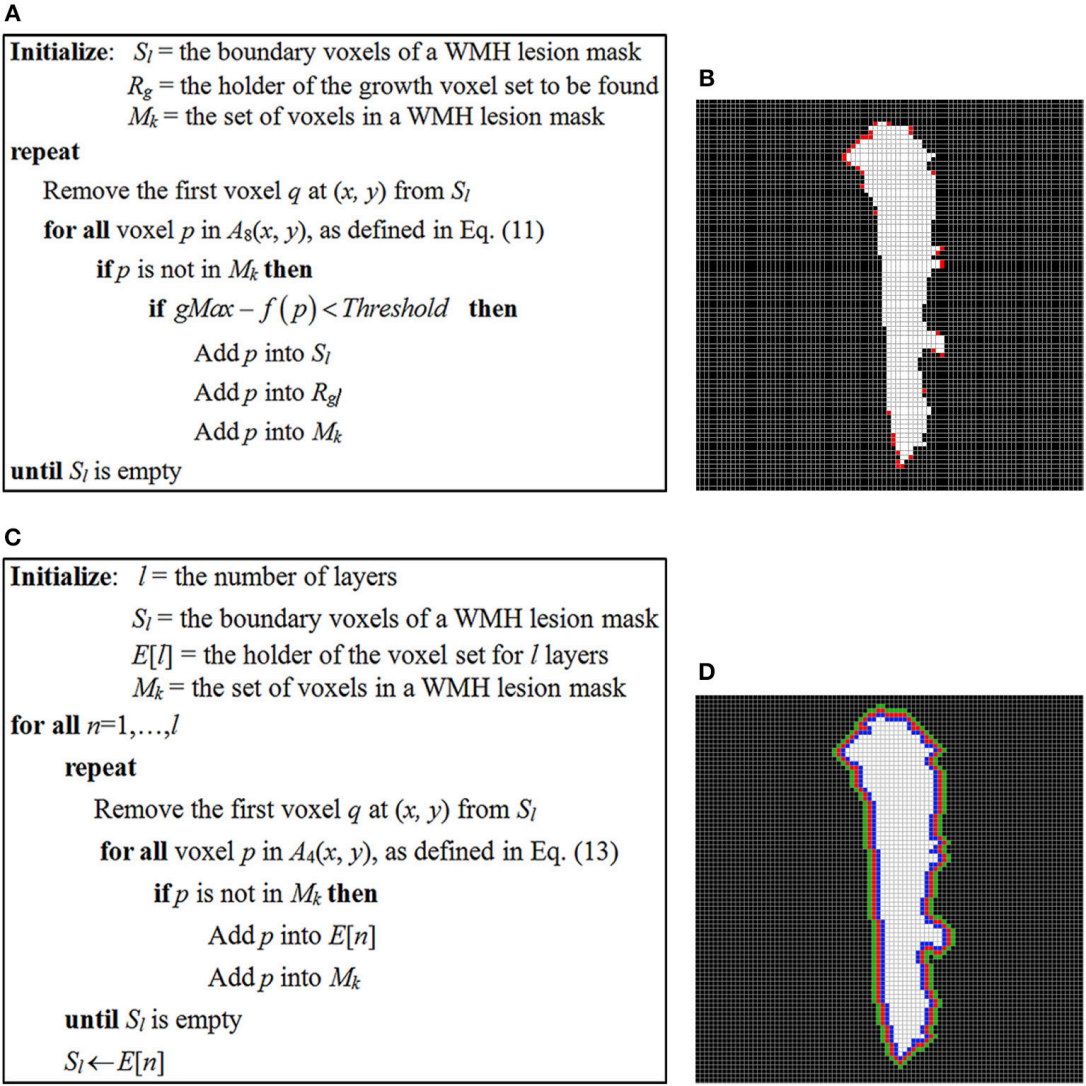
**(A)** The pseudo-code of seed-based region-growing algorithm of WMH lesions; **(B)** a WMH lesion mask and the potential growth voxels marked in red color which are identified using the seed-based region-growing algorithm. **(C)** The pseudo-code of layer generating algorithm for WMH lesions; **(D)** three one-voxel-thick layers surrounding the WMH lesion, which are used to locate a growth voxel.

In the study, the stopping criterion used for iterative seed growing is determined by comparing a neighboring voxel intensity with the highest and lowest signal intensity, *gMax* and *gMin* of a WMH3D. If the voxel intensity difference from the *gMax* is less than a threshold, as defined below in Equation (12), the corresponding voxel is designated to a growth voxel set, *R*_*g*_, and assigned to the boundary seed voxel list *S*_*l*_ for further searching. The pseudo-code of the seed-based region-growing algorithm is presented in Figure 14A. Note that *M*_*k*_ is the set of voxels in a WMH lesion mask, and *f* (*p*) is the signal intensity at neighbor voxel *p* of a lesion boundary seed voxel, as defined in Equation (11).

(12) $$\begin{array}{r} Threshold=\gamma \times \left(gMax-gMin\right) \end{array}$$

where γ is the threshold control coefficient. The choice of γ represents the user-defined steepness of the edge around the WMH boundaries. Of note, if the value of γ is too large, the potential growth region would spread around all boundaries of the WMH lesions regardless of lesion shape or texture features. In this study, we chose γ = 1.02 to demonstrate the presence of potential growth regions of WMH lesions using the seed-based growing algorithm (Figure 14B).

After all “potential growth” voxels are found, the potential growth index (PGI) of a WMH lesion can be calculated. To calculate this index, first a set of four-connected voxels [Equation (13)] to a voxel (*x, y*) on the mask boundary is applied to generate successively *l* layers of apparent masks surrounding the lesion with a layer thickness of one voxel.

(13) $$\begin{array}{r} A_{4}\left(x,y\right)=\left\{\begin{array}{c} \left(x,y-1\right),\left(x-1,y\right),\\ \left(x+1,y\right),\left(x,y+1\right) \end{array}\right\} \end{array}$$

The pseudo-code of generating *l* layers around a WMH lesion growing algorithm is presented in Figure 14C. The notations *S*_*l*_ and *M*_*k*_ are the same as in Figure 14A. *l* is the number of layers to be generated and the *i*^*th*^ layer voxels are kept in the *E*[*i*] list.

These apparent layer masks are used to identify the relative location of a growth voxel. A growth voxel at an outer layers of these masks weights more in its contribution to the potential growth index. Specifically, the weight *w*_*i*_ at *i*^th^ layer, with total *l* layers, is given by the following equation:

(14) $$\begin{array}{r} w_{i}=\frac{i}{\sum_{j=1}^{l}j} \end{array}$$

Once the number of growth voxels at each layers were calculated, the potential growth index *P*_*g*_ for each WMH lesion is calculated below:

(15) $$\begin{array}{r} P_{g}=\frac{\sum_{i=1}^{l}GV_{i}w_{i}}{V_{l}} \end{array}$$

where, *GV*_*i*_ = number of “growth voxels” found at the *i*th layer, and *V*_*l*_ = the total number of voxels in all *l* layers for a WMH.

To demonstrate the potential application, all lesion images were evaluated for their potential growth indices with *l* set to three (Figure 14D).

### The Relationship Between Potential Growth Index and WMH Shape and Texture Features

The relationship between PGI and WMH Shape and texture features was investigated in the study. The K-means algorithm is the most commonly used clustering algorithm in unsupervised learning due to its simplicity and efficiency (Hung et al., 2005), and thus is appropriate for this proof-of-concept development. However, the initial cluster seeds in K-means algorithm can generate different clustering results. To demonstrate the applicability of the K-means algorithm, we performed 1,000 trials with randomly selected initial cluster seeds from the feature vectors of shape and then texture of the lesions (a total of 993 lesions) to examine the clustering results. For the shape and texture clusters classified as shown in Figures 7, 13 above, one-way Analyses of Variance (ANOVAs) were performed to evaluate if there were significant differences in potential growth index generated from each trial among the shape or the texture clusters. Significant growth index differences for all trials were found among both shape (*P* = 2.04 × 10^−10^ to *P* = 1.06 × 10^−2^) and texture (*P* < 1 × 10^−40^ to *P* < 1 × 10^−20^) clusters. Table 1 shows the most conservative results.

**Table 1:** Potential growth indices (PGIs) for the classified shape and texture clusters.

**SHAPE**						
	**Cluster 1**	**Cluster 2**	**Cluster 3**	**Cluster 4**	**Cluster 5**	**Cluster 6**
Number of images	105	86	176	237	211	178
PGI	0.1535 ± 0.0790	0.1702 ± 0.0818	0.1376 ± 0.1051	0.1399 ± 0.0885	0.1500 ± 0.0700	0.1636 ± 0.1011
Between-cluster difference: *P* = 1.06 × 10^−2^, *F* = 3.0070						
**TEXTURE**						
	**Cluster 1**	**Cluster 2**	**Cluster 3**	**Cluster 4**	**Cluster 5**	
Number of images	128	232	265	160	208	
PGI	0.1928 ± 0.1023	0.1863 ± 0.0879	0.1037 ± 0.0683	0.1191 ± 0.0751	0.1656 ± 0.0828	
Between-cluster difference: *P* < 1 × 10^−20^, *F* = 48.4009						

*Results presented are the most conservative case from 1,000 trials with randomly selected initial cluster seeds from the feature vectors of the shape and texture of the white matter lesions*.

## Discussion and Conclusion

In this study, we have developed innovative and proof-of-concept methods to quantitatively characterize the shape (based on Zernike transformation) and texture (based on fuzzy logic) of WMH lesions. A multi-dimension feature vector approach based on these new features was used to cluster WMH lesions into distinctive groups to assess whether these features can potentially be used as image biomarkers. We have also developed an approach to calculate the potential growth index (PGI) of WMH lesions using a region-growing algorithm along the WMH boundaries. From preliminary data analyses of six subjects with a total of 993 lesions, we observed significant differences in PGI among the clustered WMH groups in terms of either the shape or the texture features. These findings, even though only a proof-of-concept, suggest that the shape and texture features of WMH can potentially be used as new imaging biomarkers to predict lesion growth in brain aging, vascular dementia, or AD.

This work demonstrates the feasibility and potential usefulness of our methods. However, there are several limitations, which are beyond the scope of this study to address completely. In WMH lesion segmentation, we adopted the mid-range point of 0.5 of the lesion probability map as the cut-off threshold suggested by the authors of the LST Toolbox. This threshold appears logical for a wide range of populations. However, different thresholds might be suitable for different study populations. Maldjian et al. (2013) suggested to use a 0.25 threshold in their study on older adults with diabetes. The change of segmentation threshold may introduce small change in the quantification of lesion sizes, which is not the focus of our work. The effects of change of segmentation threshold on characterizations of the WMH shape, texture, and potential growth should be further studied.

In shape feature extraction, all images were proportionally scaled to the same size of 60 × 60 voxels. This scaling procedure resulted in blurring the shape contours of small-size images and losing the contour details of large-size images. This one-size-fit-all scaling treatment can lead to quantification inaccuracy at higher orders of ZM. Nevertheless, we have observed that high ZM orders are not required to represent primary WMH shape features. In this study, we limit the feature characteristics at ZM ≤5. Thus, the scaling factor used in this study should have minimal effects on the shape feature extraction results. When there is a large number of WMH lesions, a more proper procedure in shape analysis can be applied to reduce the influence of this image scaling issue on shape feature extraction. Specifically, WMH lesion images can be first divided into several groups based on the size, and then are scaled appropriately based on their corresponding size groups. Shape feature analyses can then be carried in each size group. It should also be mentioned that image shape feature extraction using the Zernike transform, in theory, is independent of the image sizes to be analyzed (Teague, 1980). The purpose of image scaling in this study was to improve computational efficiency.

In texture analysis, a linear fuzzy logic method was proposed to quantize the distribution of voxel signal intensity in a lesion image. This approach is robust in handling the potential quantization error due to imaging noise (Gwo and Wei, 2013). We have chosen a linear approach in fuzzy logic and a number of bins that appeared to work well on our data. However, we have not devised a method to systematically obtain an optimal bin number or type of linear or non-linear fuzzy logic function, which needs to be investigated in studies with large sample sizes. For both shape and texture analyses, we have selected the feature dimensions that appeared reasonable to the dataset of this study. However, selecting appropriate feature dimensions and cluster numbers is still a challenging problem in the field of pattern recognition (Steinbach et al., 2004). Common approach is data-driven trial and error. For a large dataset, a supervised machine learning via artificial neural network might lead to identification of optimized feature dimensions as well as the number of group clusters (Raschka, 2015).

PGI was developed to explore the possibility of predicting WMH progression by quantification of image characteristics of WMH penumbras (Maillard et al., 2014). To do this, multiple layers surrounding a lesion mask was used to calculate PGI with a linear weighted function based on the layer locations of the “growth voxels.” The choice of a linear weighted function is consistent with the probable locations of WMH lesion development found in recent studies (Maillard et al., 2014; Promjunyakul et al., 2016). In our study, we used three layers sounding the WMH lesions to demonstrate the potential growth. A large dataset with repeated measures in longitudinal studies is needed to identify a more appropriate number of layers and devise an optimal weighting function.

We are fully aware that WMH lesion growth is likely affected by multiple factors besides the shape, texture and PGI. In this regard, the potential effects of anatomical locations of WMH on its progression rate have been investigated in prior works (DeCarli et al., 2005; Wardlaw et al., 2013). Identification of other key contributors to WMH growth and the underlying biological mechanisms are warranted for future studies.

The objective of this paper is to formulate concepts and to demonstrate the feasibility of the methods used to analyze the WMH shape, texture, and potential growth. To accomplish this object, we selected high-quality T_2_ FLAIR images which contain a large number of lesions with various sizes from six subjects as sample cases to develop our theoretical framework. While only small number of subjects was used in this study, a relatively large number of lesion (a total of 993) was used in our development and analyses. Nevertheless, the algorithms and parameters used for texture feature extraction and potential growth index estimation in this work were empirical based on trial and error, or were optimized based on the relatively small dataset. The algorithms and parameters used in this work need to be optimized based on larger datasets covering various type of lesions in future studies. Currently, we are working on the application of our methods to over 500 subjects with more than 2 years of brain imaging data from the ADNI (Alzheimer's Disease Neuroimaging Initiative) database.

Lastly, due to widely available 2D T_2_ FLAIR images in clinical practice and research, we decided to develop our concept in 2D T_2_ FLAIR first. On the other hand, we have also begun to expand our work to 3D imaging to capture the lesion shape, texture and potential growth in all spatial directions, which benefits from the recent development in 3D high-resolution T_2_ FLAIR acquisition technique (Wiggermann et al., 2016).

In summary, our work demonstrated the concept and the feasibility that shape and texture features of WMH lesions observed on T_2_ FLAIR images can be quantitatively characterized which are related to the potential growth index of white matter lesions. Future studies of large datasets and longitudinal studies based on the systematic framework proposed in this study are warranted to further optimize the algorithms and parameters used for white matter lesion shape and texture feature extraction and classification as well as PGI estimation. Furthermore, our approaches for image feature extraction and classification can potentially be generalized to other types of brain lesions and imaging modalities.

## Author Contributions

C-YG conducted experiments, wrote code to analyze the data, interpreted the data, and wrote the manuscript. DZ prepared brain images and lesion segmentation. DZ and RZ interpreted the data, participated in the scientific discussions, and provided critical insights. All authors reviewed the manuscript and approved it for publication.

### Conflict of Interest Statement

The authors declare that the research was conducted in the absence of any commercial or financial relationships that could be construed as a potential conflict of interest.
